# The effect of socioeconomic status on academic achievement: A big data study across countries and time with integrative data analysis

**DOI:** 10.1371/journal.pone.0335485

**Published:** 2025-10-31

**Authors:** Mahmut Sami Yigiter

**Affiliations:** 1 University of Salford, Salford, United Kingdom; 2 Social Sciences University of Ankara, Ankara, Türkiye; Jahangirnagar University, BANGLADESH

## Abstract

The strong relationship between socioeconomic status (SES) and academic achievement has been the focus of much research over the last fifty years. However, there is a very limited number of studies that examine the SES of individual students and the SES of their peers in a disaggregated manner. This study examines the relationship between SES and academic achievement at both the student and school levels, using data from 54 countries from six cycles of the Trends in International Mathematics and Science Study (TIMSS) assessment over the past two decades. Integrative Data Analysis (IDA) is used to synthesize data across cycles and countries, and Hierarchical Linear Modeling (HLM) is used to account for the nested nature of students within schools. The results show that both individual and school SES have a significant positive effect on academic achievement, with school SES having a stronger effect. Moreover, most of the variance in the effects of individual and school SES is due to differences between countries. Income inequality reduces the effect of individual SES and leads to differences in SES and achievement between schools. On the other hand, an increase in individual SES increases the academic achievement of female students more, while male students attending schools with higher SES levels improve their academic achievement more. Increasing the individual or school SES of migrant students helps them reduce their academic achievement gaps. Moreover, the relationship between school SES and achievement was stronger in densely populated cities and large metropolitan areas. In line with the research findings, several recommendations are made to researchers and policy makers.

## Introduction

The relationship between socioeconomic status (SES) and academic achievement has a long history [1]. The report published by Coleman et al. [2] played an important role in highlighting the effect of SES on academic achievement. SES is a multifaceted concept that encompasses an individual’s social, cultural and economic resources [3]. SES shapes individuals’ living conditions and influences their access to education, health and career opportunities [4,5]. Research in the educational context shows that SES is strongly related to academic achievement [6–8]. Students from high SES backgrounds typically have greater access to educational materials, tutoring, and academic support systems, while students from low SES backgrounds often face more limited resources [9]. Although there are many studies on the effect of students’ individual SES level on academic achievement, the number of studies that show the effect of students’ peers’ SES level on their achievement is quite limited [10,11].

Individual SES is recognised as one of the most important variables affecting students’ academic achievement [12,13]. While families’ economic power determines the extent to which they can invest in their children’s education, parents’ educational attainment shapes their ability to provide academic support to their children [14]. Parents with higher levels of education are in an advantageous position to provide their children with educational materials and resources, to encourage academic achievement and to guide learning processes [15]. On the other hand, students from low SES backgrounds face several barriers, such as family financial difficulties, inadequate educational materials and low family support for education, which may negatively affect their learning processes [3]. SES has a crucial impact on academic achievement not only at the individual level but also at the school level [16]. The fact that students attending the same school are similar in terms of SES can shape the environment in which students live and directly affect their educational opportunities [17]. High SES schools, where high SES students congregate, provide a learning environment that supports academic achievement, with a student profile focused on achievement and more educational resources provided by the SES of families [18]. On the other hand, low SES schools that cluster low SES students face several disadvantages, such as limited family resources, low motivation to learn and low academic expectations [11]. Research shows that school-level SES can have as much impact on student achievement as individual SES [19]. Although numerous studies have examined the impact of individual students’ socioeconomic status (SES) on academic achievement, research focusing on the influence of peer SES on academic outcomes remains relatively limited. The literature does include studies investigating the effect of peer SES (or school SES) on academic performance. For instance, van Ewijk and Sleegers [11] conducted a meta-analysis to examine the impact of peer socioeconomic status on academic achievement. The researchers reported a moderate effect size (d = 0.32) for peer SES on academic outcomes. Similarly, Chesters and Daly [20] explored the mediating role of peer effects in the relationship between family SES and academic achievement. Their findings highlighted that attending schools with a high proportion of students from low-educated families negatively affects individual student achievement. A more recent meta-analytic study investigating the influence of peer SES on academic achievement was conducted by Markalousová [21]. This study also found a moderate effect size (d = 0.33) for the influence of peer SES on academic performance. Overall, these studies suggest that peer SES has a significant and measurable effect on students’ academic achievement.

### Socioeconomic Status (SES)

SES is a multidimensional concept that determines the position of individuals or groups in society and is assessed using economic, social and cultural indicators [22]. It is considered a key indicator for understanding inequalities in society and the living conditions of individuals and has an impact on a wide range of areas, from individuals’ access to educational opportunities to their occupational positions, income levels and social status. This concept, which determines not only the individual development of individuals but also their mobility within social structures, stands out as a determining factor in many areas such as education, employment, health and living standards [23,24].

The main components of socioeconomic status include economic, social and cultural components [22]. The economic component includes economic indicators that directly determine the income status, assets and quality of life of the individual or household. The level of income is a factor that directly affects the ability of individuals to meet their basic needs, lead a healthy life and participate in social life [25]. The social component is related to an individual’s social relationships, occupational position and social capital. Occupational prestige, job security and the individual’s level of interaction within society are among the main factors that determine social status. The cultural component includes an individual’s level of education, intellectual capacity, cultural participation and access to information. Educational attainment is one of the most important variables in determining an individual’s position in economic and social life, increasing social mobility and broadening the opportunities available to individuals. The extent to which an individual can benefit from education and the level of access to cultural resources are also directly related to cultural capital [26].

The impact of SES is not limited to academic achievement. Many studies show that SES can have different cognitive, affective, and psychological effects on students. Studies report that students with low SES experience more stress during the educational process [27], have lower school belonging [28], are exposed to more negative judgments [29], and have lower perceptions of competence [30] than students with high SES. Another study suggests that students with higher SES have fewer emotional and behavioural problems [31]. Similarly, SES has been reported in the literature to cause cognitive, affective and psychological changes in adults. Studies report that high SES in adults increases growth mindset [32], is associated with better brain health [33,34], and is associated with higher self-esteem [35]. On the other hand, low SES is associated with more depressive symptoms and suicidal tendencies [36,37]. The results of these studies show that SES is a multifaceted concept and has diverse effects.

### Measuring SES

The variables used to measure SES are constantly changing over time as society changes [38]. Therefore, it is not possible to develop a measurement of SES that covers all ages [22]. Today, new criteria and tools for measuring SES are still being developed. As SES is thought to reflect the social position of individuals, SES is shaped by factors such as education, income, occupation, social connections and cultural capital. Therefore, SES is measured using multifaceted indicators that reflect the economic, social and cultural conditions of individuals and households [1,22]. These indicators provide important clues to understanding an individual’s standard of living, access to opportunities and social mobility.

One of the globally accepted approaches to measuring student SES is the ‘BIG 3’. The three main components of the BIG 3 are family income, parents’ educational level, and parents’ occupational status [1]. In surveys that do not include family income, income status can be related to household possessions. However, possessions may not directly reflect income, or the use of possessions over time may not be an indicator of wealth. Cowan et al. [1] suggest that assets (e.g., property, cars, etc.) can be used instead of possessions. Another problem with SES measurement is that it can vary over time, regions, countries and age groups. Therefore, temporal, regional and demographic differences should be taken into account when determining the components of SES [38–40]. In this study, the approach proposed by Broer et al. [41] was followed in order to construct a valid and comparable SES index across different TIMSS cycles.

SES is measured by combining different components in large-scale assessments such as Programme for International Student Assessment (PISA) and Trends in International Mathematics and Science Study (TIMSS) and published in the data as an index. The variables that are combined to measure SES can change between cycles. This causes problems when making comparisons with the developed indices [41]. In the TIMSS cycle, students’ responses to the Home Educational Resources Scale are used to measure SES and the Home Educational Resources (HER) index is calculated. However, as the variables in this scale and the method of calculating the index have changed over the years in TIMSS, this index cannot be used for comparisons between years [41]. For example, the HER index is calculated on the basis of the number of books, educational resources (computers, desks, dictionaries, etc.) and parents’ educational level (three levels: high, medium, low) in 1995 and 1999. The question on Internet access was not included in 2007, but was included in this scale in subsequent cycles. Similarly, computer ownership has been split into two variables as individual ownership and shared use since the 2015 cycle. On the other hand, the calculation method of the index was changed from 2011 onwards and started to be scaled on the basis of Item Response Theory – IRT. All these changes make it difficult to make direct comparisons when comparing the HER index obtained from TIMSS [41]. In this study, students’ SES scores were obtained as suggested by Broer et al. [41].

### Literature review

There are studies in the literature that examine the effect of student SES on academic achievement. In his meta-analysis study, Sirin [7] reported the average effect size of 58 articles published between 1990 and 2000 as 0.27. Koza Çiftçi and Cin [42], in a meta-analysis study that included 66 primary research findings, reported a high (0.41) effect of student SES on academic achievement. Liu et al. [6] combined the studies from the Chinese sample between 1989 and 2016 with a meta-analysis and reported that student SES had an average effect size of 0.24 on academic achievement. Kim et al. [43] combined 49 experimental studies published between 1990 and 2017 with meta-analysis and calculated the effect size to be 0.12. On the other hand, there are also studies in the literature that examine the effect of school SES level on academic achievement. For example, van [11] examined the effect of the socio-economic status of peers on academic achievement using meta-analysis. The researchers calculated the average effect size of peer SES on academic achievement to be 0.32, and found that peer SES may be an important determinant of academic achievement. Xuan et al [44] examined the effect of school SES on mathematics and Chinese achievement, and found that school SES had a significant effect on both types of achievement. In addition, the researchers reported that the student-teacher relationship partially mediated the effect of school SES on achievement. Studies examining the effect of both student and school SES on academic achievement are rather limited. Caldas and Bankston [45] examined the effects of student SES and peer (school) SES on academic achievement in the same model and reported that student SES had a significant effect on academic achievement and that peer SES had a significant and important effect on academic achievement, although not as significant as student SES. Finch and Hernández Finch [46], in their study comparing the effects of student SES and school SES on mathematics achievement, find that student SES and school SES have a significant effect on academic achievement. The study also reports that the effect of school SES is greater than the effect of student SES. The researchers state that student achievement can be raised in a balanced way by reducing inequalities and increasing public resources. Coşkun and Karakaya Özyer [47] examined the effect of school SES on mathematics achievement using HLM and included many variables in the model. The results of this study show that school SES is the variable that most affects mathematics achievement. Similarly, Gustafsson et al [48] examined student achievement with a two-level HLM model and found that School SES is the strongest determinant of achievement differences. Ersan and Rodriguez [49] examined the effect of student SES and school SES on academic achievement using TIMSS 2015 Turkish data. In this study, the researchers report that both student SES and school SES are dominant factors in academic achievement. As can be seen, many studies have been conducted on the impact of SES on academic achievement. While the majority of these studies have examined the effect of student SES on academic achievement, some of them have also examined the effect of school SES on academic achievement. The number of studies examining the effect of both student SES and school SES on academic achievement is rather limited.

### Research Aim and Significant

This study aims to examine the socioeconomic factors that affect students’ academic achievement not only at the individual level, but also through SES of their peers in the school environment. This variable, defined as school SES, is based on the average SES of other students in the school rather than on the student’s own SES. This perspective provides a new insight into the indirect effects of the social and economic environment within the school on student achievement. SES is recognised as one of the main determinants of educational inequalities. However, this effect, usually considered at the individual level, combines with peer effects in the school environment to create a more complex structure. This study examines the collective impact of the socioeconomic structure of schools on student achievement, by examining how the academic achievement of students whose peers are of higher or lower SES differs. Socioeconomic status is an important factor that shapes student achievement not only individually, but also through the social environment within the school. This approach contributes to the development of more equitable and inclusive education strategies by providing a new perspective in designing policies to ensure equity and opportunity in education. This study examines the effects of student and school SES on academic achievement in 54 different countries, using data from the last 20 years of the TIMSS large-scale assessment. The study aims to understand how the school environment shapes student achievement in different socioeconomic contexts around the world. A comparative analysis of data from 54 countries shows how education systems and socioeconomic conditions in different countries differentiate this relationship. This international perspective highlights that the school SES has profound implications for education policy and the understanding of equity not only in the local context, but also on a global scale. Furthermore, similarities and differences between student and school SES are analysed by country, continent and moderator variables, as well as the international Gini index.

Within the scope of the research, answers to the following three questions were sought:

1) What is the effect of Student SES and School SES on academic achievement?
2) Do the effects of Student SES and School SES on academic achievement differ according to moderator variables?
3) Do the effects of Student SES and School SES on academic achievement differ according to income inequality?

## Method

### Data Source

In this study, data from the 2003, 2007, 2011, 2015, 2019 and 2023 TIMSS cycles from 54 countries and worldwide over 20 years were used for analysis to examine the impact of student SES and school SES on academic achievement. The main purpose of the TIMSS studies is to assess the mathematics and science achievement of students in grades 4 and 8 in different countries and to provide regular internationally comparable data on the performance of education systems. In addition to the achievement tests, TIMSS collects a wide range of other information about the students themselves and their families. One reason for choosing the TIMSS cycle to examine the impact of student and school SES on academic achievement is the difficulty of measuring SES comparably across cultures and years. There are different SES indices in large-scale assessments, but these indices may be calculated with different items in different cycles. When SES indices calculated in this way are compared across cultures and time periods, the interpretation of the statistics obtained will also differ. Therefore, the SES index developed by Broer et al. [41] for the TIMSS cycles was used in this study. The data used in the study were obtained from the TIMSS database (https://timssandpirls.bc.edu/databases-landing.html).

### Samples

The sample of this study consists of 1,138,631 8th grade students who participated in the TIMSS 2003, 2007, 2011, 2015, 2019 and 2023 cycles. In the TIMSS cycles, data from grade 4 were not included in the study. The reason for this is the lack of data necessary to calculate the SES index proposed by Broer et al. [41] at the TIMSS 4th grade level. Therefore, the study was conducted with 8th grade students in TIMSS cycles. 54 countries were included in the study. While 19 of these countries participated in all 6 cycles, 12 participated in 5 cycles, 8 in 4 cycles, 7 in 3 cycles and 8 in 2 cycles. Countries that participated in only one of the TIMSS cycles were not included in the study. The number of countries, schools and students according to the cycles included in the study is shown in Table 1.

**Table 1:** Number of countries, schools and students by cycle.

Cycle	Country		School		Student	
	n	%	N	%	n	%
2003	41	15,36	7608	19,61	272520	23,93
2007	45	16,85	6999	18,04	197034	17,30
2011	44	16,48	6549	16,88	209601	18,41
2015	48	17,98	6845	17,65	173839	15,27
2019	48	17,98	5811	14,98	160348	14,08
2023	41	15,36	4979	12,84	125289	11,00
Total	267	100,00	38791	100,00	1138631	100,00

In summary, 272520 students from 41 countries from the TIMSS 2003 cycle, 197034 students from 45 countries from the TIMSS 2007 cycle, 209601 students from 44 countries from the TIMSS 2011 cycle, 173839 students from 48 countries from the TIMSS 2015 cycle, 160348 students from 48 countries from the TIMSS 2019 cycle, 125289 students from 41 countries from the TIMSS 2023 cycle, and 1138631 students in total formed the sample of this study.

### Measures

#### Student socioeconomic status (Student SES).

In constructing the SES variable, the steps suggested by Broer et al. [41] were followed. Broer et al. [41] state that the SES variable can be obtained by summing the sub-variables of parents’ highest level of education (bsdgedup), computer/tablet ownership (bsbg05a), desk ownership (bsbg05b), and number of books (bsbg04) in the TIMSS student questionnaire to obtain a score between 0–10, and this score can be used as the SES index. All six cycles in this study included all of these sub-variables.

#### School socioeconomic status (School SES).

Cowan et al. [1] emphasise that the SES level of a school can be obtained by summing the SES levels of the students attending that school. In constructing this variable, the SES levels of other students in each student’s school were collected and divided by the number of students. Therefore, this variable shows the average SES level of a student’s peers.

#### Achievement scores.

In large-scale assessments such as TIMSS, the booklet matrix sampling method is used to measure achievement, rather than administering the same tests to all students. In this method, test items are divided into multiple booklets and randomly assigned to students. As a result, students answer only a portion of the questions in the item pool, creating a large matrix of missing data in the dataset. The incomplete data matrix makes it difficult to predict students’ academic achievement, so instead of predicting a single achievement score, multiple plausible values (PVs) are predicted by statistical models. Thus, instead of one achievement score for each student, multiple PVs representing academic achievement are generated. In this study, PVs from all TIMSS cycles were used as academic achievement scores. The IEA provides 5 PVs in each TIMSS cycle and for each domain. In this study, the PVs for mathematics and science were used as achievement scores. Academic achievement was modelled using the IMPUTATION method proposed for the use of these scores [50]. In addition, the PV values of the mathematics subdomains of Numbers, Algebra, Geometry and Data and Probability and the Science subdomains of Biology, Chemistry, Physics were also included in the subgroup analyses. Finally, the PV values of Knowing, Applying and Reasoning scores from the Cognitive Domain were also included in the analyses as sub-variables.

### Subgroups variables

In this study, there are 12 variables from the TIMSS student and school questionnaires whose moderating effects are examined. These variables were generally derived from the responses of participating students to the TIMSS student questionnaire or from the responses of participating school administrators to the TIMSS school questionnaire. In constructing the variables, variables were selected that were present in all six cycles included in the study. The names and subcategories of the variables may change over the years, and in order to make accurate comparisons across years, the researchers renamed the variables with codes in R to create all cycles in a similar way. The variables included in the TIMSS 2019 cycle questionnaire and the variable names are described below.

***Gender*** It is the variable where students indicate their gender (Are you a girl or a boy?). In all cycles, it is coded as Girl = 0 and Boy = 1. In the TIMSS cycle, this variable is coded as “itsex”.

***Language Spoken at Home***: The variable in which students indicate to what extent they speak the language of the test at home (How often do you speak <language of test> at home?). In the TIMSS cycle, this variable is divided into four categories (Always = 4, Almost always = 3, Sometimes = 2, Never = 1) and is included in TIMSS 2019 with the code “BSBG03”.

***Education Goal*** It is the variable where students indicate the level at which they expect to progress in education (How far in your education do you expect to go?). This variable is divided into 6 categories in the TIMSS cycle, but in this study it is coded as 5 categories since not all cycles have the same categories (Finish <Lower secondary education = 1, Finish <Upper secondary education = 2, Finish <Post-secondary, non-tertiary education = 3, Finish <Short-cycle tertiary education = 4, Finish <Bachelor’s or equivalent level = 5, Finish <Postgraduate degree: Master or Doctor = 5). This variable is included in the TIMSS 2019 cycle with the code “bsbg07”.

***Mother’s Country of Birth*** The variable where students indicate the place of birth of their mother (or Parent A) (Were your <parents/guardians> born in <country > ?). This variable is included in the TIMSS 2019 cycle with the code “bsbg08a”. This variable has two categories (Yes = 1, No = 0).

***Father’s Country of Birth*** The variable where students indicate the place of birth of their father (or Parent B) (Were your <parents/guardians> born in <country > ?). This variable is included in the TIMSS 2019 cycle as “bsbg08b”. This variable has two categories (Yes = 1, No = 0).

***Student’s Country of Birth*** It is the variable where students indicate their place of birth (Were you born in?). This variable is included in the TIMSS 2019 cycle as “bsbg09a”. This variable has two categories (Yes = 1, No = 0).

***Population of School District*** The variable where school administrators indicate the number of people living in the area where your school is located (How many people live in the city, town, or area where your school is located?). This variable is included in the TIMSS 2019 cycle under the name “bcbg05a”. This variable has seven categories (“more than 500,000 people” = 7, “100,001 to 500,000 people” = 6, “50,001 to 100,000 people” = 5, “30,001 to 50,000 people” = 4, “15,001 to 30,000 people” = 3, “3,001 to 15,000 people” = 2, “3,000 people or fewer” = 1).

**School Location** It is the variable where school administrators indicate the type of settlement where the school is located (Which best describes the immediate area in which your school is located?). This variable is included in the TIMSS 2019 cycle with the code “bcbg05b”. This variable has five subcategories (“urban-densely populated” = 5, “suburban-on the fringe or outskirts of urban area” = 4, “medium-sized city or large town” = 3, “small town or village” = 2, “remote rural” = 1).

**Content Domain** It refers to the subject categories identified in the TIMSS cycles to measure students’ knowledge and skills in mathematics and science assessments. In the 2019 TIMSS cycle, mathematics has four content domains: Number (bsmnum0), Algebra (bsmalg0), Geometry (bsmgeo0), Data and Probability (bsmdat0). Science also has four content domains: Biology (bssbio0), Chemistry (bssche0), Physics (bsssphy0), Earth Science (bssear0). In the study, all content domains were included in the model of the effect of student SES and school SES on achievement, effect sizes were calculated and their effects analysed.

**Cognitive Domain** It is a framework that assesses how students process, apply and analyse knowledge and skills in the mathematics and science domains in the TIMSS cycles. The cognitive domain is divided into three basic categories to measure students’ level of knowledge and how they use that knowledge: Knowing (Basic Knowledge - bsmkno0), Applying (Using Knowledge - bsmapp0) and Reasoning (Reasoning - bsmrea0). In the study of the effect of student SES and school SES on achievement, all content areas were included in the model and effect sizes were calculated and compared.

**Gini-Index** The Gini index is a statistical indicator that measures inequality in the distribution of income or wealth in countries. It was developed by the statistician Corrado Gini. This index usually takes a value between 0 and 1. A value of 0 indicates that there is complete equality in society, i.e., everyone has the same income, while a value of 1 indicates that income is entirely concentrated in one person, i.e., maximum inequality. The data for this index come from the World Bank (https://data.worldbank.org/indicator/SI.POV.GINI). Please note that data for certain years are missing for some of the countries included in the dataset. To address the issue of missing data, first, pairwise correlations between years were examined. The average correlation between years was calculated as r = 0.94 (SD = 0.03), indicating a high degree of similarity in countries’ Gini indices over time. Therefore, missing values were imputed using the country-level mean.

### Data analysis approach

Integrative data analysis was used to investigate the impact of student SES and school SES on academic achievement [51]. In this approach, the large dataset is divided into smaller datasets according to clusters within the large dataset, each of the smaller datasets is analysed separately, and then the findings from the meta-analytic approach are combined. This approach reduces methodological bias between primary studies [52,53]. Recently, Campos and Scherer [54], Pietsch et al. [55] and Keller et al. [56] have adapted this approach to ILSAs. The first and most important advantage of applying the IDA approach to ILSA data is that ILSAs are rigorous in sample selection and the sample is highly representative of the country. In this way, sample selection bias is reduced and effect sizes can be more accurately determined [52]. In addition, the fact that ILSAs use the same questionnaires across years and countries reduces the bias and heterogeneity caused by different questionnaires in classical single-stage meta-analyses [54]. The integrative data analysis was carried out in two stages. In the first stage, effect sizes were calculated on a country basis. In the second step, the effect sizes were combined.

### Stage 1: Calculating effect sizes

Data from the 2003, 2007, 2011, 2015, 2019, 2023 TIMSS cycles were obtained. The first step was to analyse the missing data. The rate of missing data for the four variables used to construct the SES variable ranged from 1.9% to 3.1%. Due to the low rate of missing data, missing data were imputed using the EM algorithm, one of the advanced missing data imputation methods. No missing data were found for the plausible values (PVs) used as achievement scores. A two-stage hierarchical linear model (HLM) was constructed for each country. The formula for the HLM model was as follows:

1 Student-Level (Level-1) Equation:

$${Achievement}_{ij}=\beta_{\text{0}j}+\beta_{\text{1}j}({Stu\_SES}_{ij})\;+\;\beta_{\text{2}j}({Sch\_SES}_{ij})+e_{ij}$$

- ${Achievement}_{ij}$: Achievement score of student i in school j.
- $\beta_{\text{0}j}$: Intercept for school j (average achievement in school j).
- $\beta_{\text{1}j}$: Effect of student SES on achievement.
- $\beta_{\text{2}j}$: Effect of school SES on achievement.
- $e_{ij}$: Individual-level error term (random variation at the student level).

2 School-Level (Level-2) Equations:

$$\beta_{\text{0}j}=\gamma_{\text{0}\text{0}}+u_{\text{0}j}$$

- $\gamma_{\text{0}\text{0}}$: Overall average achievement across all schools.
- $u_{\text{0}j}$: Random effect capturing between-school variability in average achievement.

A two-level HLM regression model was constructed with student SES and school SES as predictor variables, and academic achievement scores (mathematics and science achievement scores) as the first-level outcome variable. Before calculating the regression coefficients, centering was performed to separate the effects of student SES and school SES. The student SES variable was centered using the group mean centering method, in which each student’s SES score was subtracted from the average SES score of the school to which the student belonged, to clarify the student’s position within the school. The school SES was centred using the grand mean centering method. By subtracting the overall average of all schools from each school SES value, the socioeconomic status of the school was assessed more accurately than the overall national average. These practices clarify the relationship between the effects of individual-level variables and the effects of group-level variables, and allow for accurate analysis of cross-level effects [57]. In addition, this process minimises distortions and errors in the model and increases the precision of the group-level fixed effects [58].The decomposition of the effects of the variables in the model reduces the estimation errors of the model and increases its reliability [59]. Student academic achievement scores (mathematics and science), student SES and school SES scores were then standardised around the mean for each country (mean = 0.00, sd = 1.00). Standardisation made the regression parameters between student and school SES and academic achievement comparable and reduced multicollinearity [60]. The VIF (variance inflation factor) value was used to check whether there was a problem of multicollinearity between the independent variables. The VIF values were very close to 1 in all countries. It was therefore decided that there was no multicollinearity. Plausible values (PV) were used to assess academic achievement. Plausible values (PVs) are used in large-scale assessments (PISA, TIMSS, PIAAC) to provide more accurate estimates at the population level, rather than directly calculating individual student scores. In large-scale assessments, students are given different booklets, resulting in an incomplete data matrix. Since not all students answer the same questions, the missing data is filled in using item response theory (IRT) and Bayesian approaches to generate multiple (usually 5 or 10) possible scores for each student. This method reduces measurement error and makes group-level analyses more reliable [61]. PVs are not used to show the exact scores of individuals, but rather to accurately calculate standard errors in further analyses such as population-wide statistical analyses and multi-level modelling. To avoid incorrect results, all PVs should be used in the analyses instead of a single PV. The literature recommends using PVs as achievement scores in combination with the imputation method [50,62]. The imputation approach reveals students’ achievement levels with multiple estimates representing different probabilities instead of a single value, thus increasing statistical precision while solving the problem of missing data. PV is often combined with multiple imputation techniques and allows for more reliable analyses by accounting for uncertainties in the data. In this study, PV scores were used as achievement scores using the imputation method. Because international assessment studies such as TIMSS have complex sampling designs, it is important to use sample weights in statistical analyses. While the appropriate use of weights in HLM analyses increases the accuracy and generalizability of model results, incorrect application can lead to biased estimates, miscalculated standard errors, and reliability issues [63]. It is therefore recommended that analyses conducted with TIMSS data consider both normalized total weights at the student level and school weights at the school level. In this study, sample weights were incorporated into the HLM model as student-level total weights (totwgt) and school-level school weights (schwgt). Following the completion of these steps, the study proceeded to calculate standardized regression coefficients separately for all cycles and all countries (see S1 Appendix).

### Stage 2: Merging Effect Sizes

**Fig 1:**
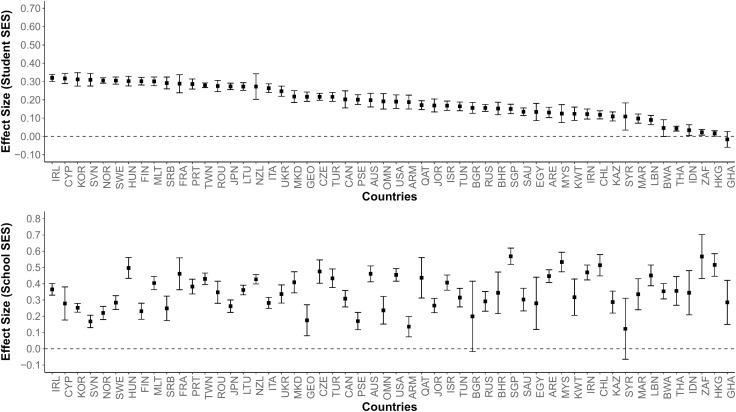
Effect Sizes by Country (Mathematics Achievement). *Note.* Numbers represent effect sizes obtained from standardized regression coefficients.

**Fig 2:**
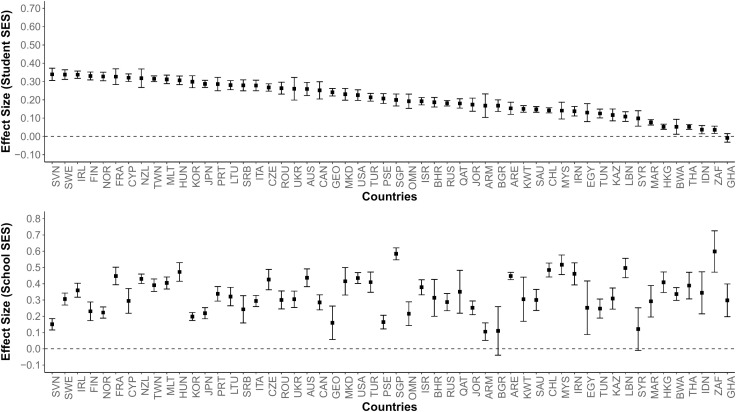
Effect Sizes by Country (Science Achievement). *Note.* Numbers represent effect sizes obtained from standardized regression coefficients.


In the second stage, the standardized regression coefficients obtained from the cycles and countries were combined with the multilevel meta-analytic approach to calculate the impact of student SES and school SES on academic achievement (see Table 1 for details). Additionally, the standardized regression coefficients of the countries obtained from all cycles were combined with the multilevel meta-analytic approach to compare the effects of student SES and school SES (see Figs 1 and 2). The number of TIMSS cycle participants from countries was used as a weight in combining effect sizes [64]. The effect sizes were nested: cycle, country and primary level. Consequently, a three-level multilevel meta-analytic model was developed to calculate a common effect size from the nested effect sizes. The model is given below [65]:

$${\text{ First level }y}_{ij}\;=\;\lambda_{ij}\;+\;e_{ij}$$

$${\text{Second level }\lambda }_{ij}\;=\;\beta_{\text{0}j}\;+\;u_{ij}$$

$${\text{ Third level }\beta }_{\text{0}j}\;=\;\gamma_{\text{0}\text{0}}\;+\;u_{\text{0}j}$$

$$\text{ Combined formula }y_{ij}\;=\;\gamma_{\text{0}\text{0}}\;+\;u_{\text{0}j}\;{+\;u}_{ij}\;+\;e_{ij}$$

In the first level of the model, $y_{ij}$ is the i-th effect size in country j,$\;\lambda_{ij}$ is the expected effect size of the study, and $e_{ij}$ is the residual error term. At the second level, the average effect size in country j is denoted by $\beta_{\text{0}j}$, while $u_{ij}$ is the random variation due to studies within the country. Finally, at the third level, the overall average effect size is represented by $\gamma_{\text{0}\text{0}}$, while $u_{\text{0}j}$ is defined as the random variation across years.

The reasons for the difference between the effect sizes of Student SES and School SES on academic achievement were examined through subgroup analyses. Subgroup analyses of continuous variables were conducted by incorporating the interaction effect in the model, as recommended by Bruner et al. [52]. Effect size differences between groups of categorical variables were analysed by meta-analytic moderator analysis. Initially, the effect sizes of the interaction effect (e.g., SES * Gender) incorporated within the model were determined using standardized regression coefficients derived from the model across all cycles and countries. Subsequently, the standardized regression coefficients were integrated with the meta-analytic approach, thereby yielding the joint effect size. It is important to note that the Gini index, which is typically employed at the country and year levels, was not a student- or school-level variable in this study. Consequently, the relationship between the Gini index and the effect sizes of student SES and school SES was analysed and reported using meta regression [64].

All analyses were performed in R. “EdSurvey” for TIMSS data collection [66], “mvdalab” for missing data assignment [67], “dplyr” [68] for data manipulation, “WeMix” [69] for HLM analysis, “metaphor” [70] for meta-analytic analysis, “ggplot2” [71] and “ggrepel” [72] packages for data visualization. All codes and data in the study are openly published at https://osf.io/qkpnc/.

## Results

From the TIMSS 2003, 2007, 2011, 2015, 2019 and 2023 cycles and 54 countries included in the study, 267 standardized regression coefficients were obtained for Student SES and 267 for School SES with HLM.

### Effects of Student SES and School SES on academic achievement

The multivariate meta-analytic random effects model demonstrates that student SES has a moderate effect on mathematics achievement (B = 0.18, 95% CI [0.16, 0.20]) and school SES has a moderate effect on mathematics achievement (B = 0.37, 95% CI [0.34, 0.40]). A similar pattern is observed for science achievement, with student SES showing a moderate effect (B = 0.20, 95% CI [0.18, 0.22]) and school SES demonstrating a moderate effect (B = 0.36, 95% CI [0.33, 0.39]).

The effect of student SES on math achievement (B = 0.18, 95% CI [0.16, 0.20]) is smaller than the effect on science achievement (B = 0.20, 95% CI [0.18, 0.22]). On the other hand, the effect of school SES on math achievement (B = 0.37, 95% CI [0.34, 0.40]) was higher than the effect of school SES on science achievement (B = 0.36, 95% CI [0.33, 0.39]). These findings suggest that school SES is more important for math achievement and student SES is more important for science achievement. The heterogeneity levels of the common effect sizes obtained with the multilevel meta-analytic approach were calculated for each level with Q and $I^{\text{2}}$ statistics (see Table 3) (Van den Noortgate et al., 2015).

**Table 3:** Heterogeneity Tests.

Achievement	Variable	Q	df	p	I^2^ Level-1	I^2^ Level-2	I^2^ Level-3
Mathematics	Student SES	8459,9	266	0,000	3,18	96,82	0,00
	School SES	2848,9	266	0,000	9,66	88,62	1,73
Science	Student SES	8047,0	266	0,000	3,20	95,78	1,02
	School SES	2929,2	266	0,000	8,99	87,64	3,37

As demonstrated in Table 2, the heterogeneity value due to sampling error in I2 Level-1 in the effect of student SES on mathematics achievement is 3.2%. However, the intra-cluster heterogeneity percentage in the I2 Level-2 cell is quite high at 96.8%. Inter-cycle heterogeneity in the I2 Level-3 cell is 0.0%. These findings indicate that heterogeneity across years is minimal, with the majority of the observed heterogeneity (96.8%) due to differences between countries. In the context of the investigation into the effect of school SES on mathematics achievement, while the heterogeneity due to sampling error is 9.7% at I2 level 1, the percentage of intra-cluster heterogeneity in the I2 level 2 cell is quite high at 88.6%. Inter-cycle heterogeneity in the I2 Level-3 cell is 1.7%. It is seen that cross-country differences (88.6%) are also a significant source of heterogeneity in the effect of school SES on mathematics achievement. The findings of the heterogeneity analysis are also statistically significant (p < .001).

**Table 2:** Effect size according to random effects model.

Achievement	Variable	N	Effect Size	Standard Error	*z*	*p*	95% Confidence Interval	
							Lower	Upper
Mathematics	Student SES	267	0,180	0,008	22,1	0,000	0,164	0,195
	School_SES	267	0,373	0,013	28,0	0,000	0,347	0,400
Science	Student SES	267	0,197	0,009	21,5	0,000	0,180	0,215
	School_SES	267	0,358	0,015	23,7	0,000	0,329	0,388

*Note.* Student SES: Student Socio Economic Status, School SES: School Socio Economic Status.

The findings indicate that the majority of observed heterogeneity is due to differences between countries. The differences in the effect sizes of student SES and school SES on academic achievement by country are shown in Fig 1 (mathematics) and Fig 2 (science). In both figures, student SES only is ranked in descending order of effect size, while the position of the countries is kept constant for better comparisons between countries. Country names are taken from ISO (ISO 3166−1 alpha-3) codes (https://en.wikipedia.org/wiki/ISO_3166-1_alpha-3). Country names, average effect sizes, confidence intervals, and Z and p values are available in S1 Appendix.

As demonstrated in Figs 1 and 2, the majority of countries with the largest effects of student SES on achievement are located in Europe (e.g., Hungary, Romania, Ireland, Serbia, Sweden, Slovenia, Portugal, Norway, France, etc.). On the other hand, the countries where school SES has the largest effect on achievement are generally located in Africa (South Africa, Ghana, Botswana, etc.) and Asia (Indonesia, Hong Kong, Singapore, Lebanon, etc.). This finding suggests that Student SES and School SES levels may differ according to the continent in which the countries are located, as well as the difference arising from the countries themselves.

**Fig 3:**
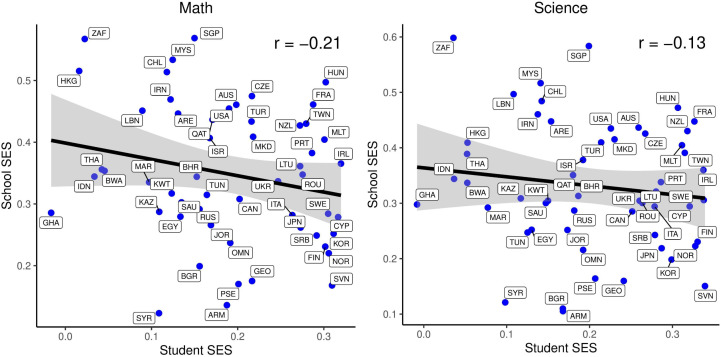
The Relationship between the Effect Sizes of Student SES and School SES.

Furthermore, an analysis was conducted to investigate the relationship between the student SES and the school SES effect size of the countries. The findings revealed a general trend where countries with high Student SES effect size exhibited low School SES effect size, and conversely, countries with high School SES effect size demonstrated low Student SES effect size. The effect sizes of Student SES and School SES are presented on a graph by country (Fig 3).

As demonstrated in Fig 3, a negative correlation is evident between student socio-economic status (SES) and school SES, in both mathematics and science achievement. Specifically, the correlation value for mathematics achievement is r = −0.21 (p < 0.001), while for science achievement, it is r = −0.13 (p < 0.001). This finding indicates that in countries where the effect of student SES on academic achievement is high, the effect of school SES on academic achievement is generally low. This finding suggests a reciprocal relationship, whereby an increase in the effect of individual SES on academic achievement is accompanied by a decrease in the effect of school SES on academic achievement. In other words, in countries where individual SES is more important, the impact of school SES is lower. A further analysis of the data reveals that in countries occupying the upper left quadrant of the graphs (e.g., South Africa, Hong Kong, Singapore, Lebanon, Malaysia), the impact of school SES on academic achievement is higher, while the impact of student SES is lower. This suggests that, in these countries, the socioeconomic structure of the school environment exerts a greater influence on academic achievement than the individual socioeconomic status of the student. Conversely, in the lower right corner of the graphs (e.g., Slovenia, Serbia, Korea, Norway, Finland), the impact of individual SES on academic achievement was higher, while the impact of school SES was lower. This finding suggests that individual socioeconomic conditions exert a more substantial influence on educational outcomes. In contrast, countries situated in the middle of the graphs (e.g., Turkey, Japan, Lithuania, Portugal) exhibit a moderate balance between the effects of student SES and school SES.

### Subgroup analysis

#### Subgroup analysis of continuous variables.

**Fig 4:**
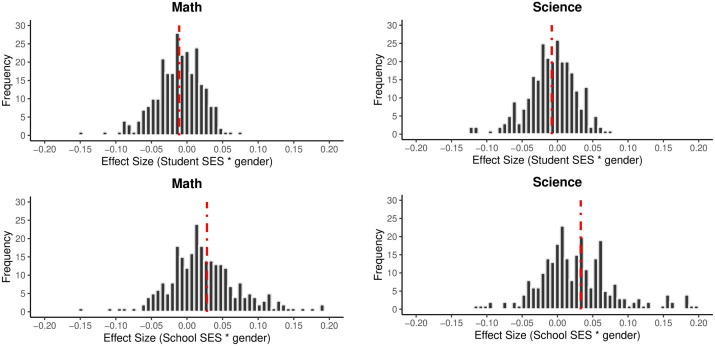
Effect Sizes of Student SES and School SES by Gender. *Note.* The red dashed line indicates the average effect size.

Subgroup analyses of continuous variables are presented in Table 4. The effect sizes in Table 4 were calculated by adding the interaction effect of the variable to the model (e.g., Student SES * Gender). It is seen that the effect of gender and the effects of Student SES and School SES on academic achievement are quite different. While the effect of Student SES on academic achievement (Mathematics: B = −0.01, p < 0.001; Science: B = 0.01, p < 0.001) is in favor of female students, the effect of School SES on academic achievement (Mathematics: B = 0.03, p < 0.001; Science: B = 0.03, p < 0.001) is in favor of male students. In other words, while the increase in the Student SES level of female students increases their academic achievement more than male students, the fact that male students are educated in schools with high level school SES increases their academic achievement more than female students. Fig 4 shows the effect sizes obtained from all cycles and countries separately for science and mathematics achievement.

**Table 4:** Subgroup analysis of continuous variables.

Achievement	Variable	Student Socio Economic Status (Student SES)									School Socio Economic Status (School SES)						
		N	Effect Size	se	Z	df	p	LB	UB	N	Effect Size	se	Z	df	p	LB	UB
Mathematics	Gender	267	−0,011	0,002	−5,479	1	0,000	−0,015	−0,007	267	0,028	0,003	8,453	1	0,000	0,022	0,035
	Language Spoken at Home	266	−0,002	0,001	−1,572	1	0,116	−0,005	0,001	266	0,007	0,002	4,636	1	0,000	0,004	0,010
	Education Goal	267	0,021	0,002	12,130	1	0,000	0,018	0,025	267	0,016	0,002	8,545	1	0,000	0,012	0,020
	Mother’s Country of Birth	267	0,008	0,008	0,980	1	0,327	−0,008	0,023	267	−0,016	0,007	−2,307	1	0,021	−0,029	−0,002
	Father’s Country of Birth	267	0,012	0,007	1,751	1	0,080	−0,001	0,026	267	−0,026	0,015	−1,718	1	0,086	−0,055	0,004
	Student’s Country of Birth	267	−0,014	0,007	−1,917	1	0,050	−0,028	0,000	267	−0,036	0,012	−3,015	1	0,003	−0,059	−0,012
	Population of School District	221	0,001	0,001	1,172	1	0,241	−0,001	0,004	221	0,043	0,005	8,478	1	0,000	0,033	0,053
	School Location	173	0,003	0,002	1,973	1	0,052	0,000	0,006	172	0,038	0,005	7,557	1	0,000	0,028	0,048
Science	Gender	267	−0,008	0,002	−3,999	1	0,000	−0,013	−0,004	267	0,033	0,005	7,199	1	0,000	0,024	0,042
	Language Spoken at Home	266	0,001	0,001	0,602	1	0,547	−0,002	0,003	266	0,010	0,002	5,945	1	0,000	0,007	0,013
	Education Goal	267	0,021	0,002	11,828	1	0,000	0,018	0,025	267	0,010	0,002	5,922	1	0,000	0,007	0,014
	Mother’s Country of Birth	267	−0,003	0,007	−0,360	1	0,719	−0,017	0,012	267	−0,022	0,008	−2,853	1	0,004	−0,037	−0,007
	Father’s Country of Birth	267	0,009	0,007	1,323	1	0,186	−0,005	0,023	267	−0,048	0,011	−4,436	1	0,000	−0,069	−0,027
	Student’s Country of Birth	267	−0,023	0,007	−3,144	1	0,002	−0,038	−0,009	267	−0,039	0,011	−3,674	1	0,000	−0,060	−0,018
	Population of School District	221	0,003	0,001	2,599	1	0,009	0,001	0,006	221	0,036	0,005	7,277	1	0,000	0,026	0,046
	School Location	173	0,005	0,002	2,935	1	0,003	0,002	0,009	172	0,030	0,005	6,223	1	0,000	0,021	0,040

*Note.* N: Number of Effect Size, se: standard error, LB: lower bound, UB: upper bound.

The negative effect size of the student SES * gender interaction indicates that the effect of student SES on academic achievement is weaker for male students. Conversely, this suggests that as Student SES increases, the academic achievement of female students may exhibit a greater increase than that of male students. On the other hand, the positive effect size of the school SES * gender interaction demonstrates that the socioeconomic level of the school has a more significant effect on the achievement of male students. This suggests that there is a more significant increase in the academic achievement of male students studying in schools with higher SES levels.

Upon analysis of the interaction effect of Language Spoken at Home, it was found that the effect of Student SES on academic achievement was statistically insignificant (Mathematics: B = 0.00, p > 0.05; Science: B = 0.00, p > 0.05). However, the effect of school SES on academic achievement was found to be significant (Mathematics: B = 0.01, p < 0.001; Science: B = 0.01, p < 0.001). Despite the statistically significant effect size of the School SES * Language Spoken at Home interaction, it is relatively negligible, approaching zero.

**Fig 5:**
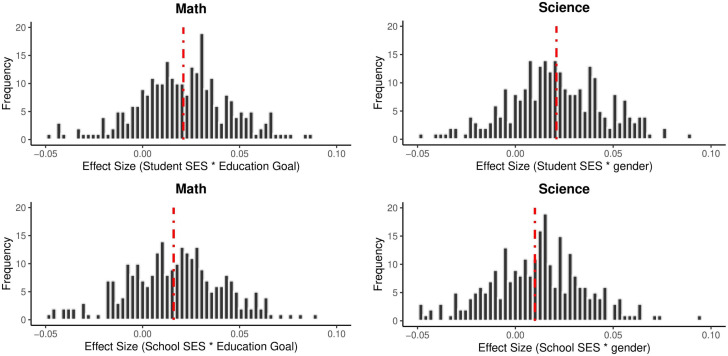
Effect Sizes of Student SES and School SES by Education Goal. Note. The red dashed line indicates the average effect size.

When analysing the interaction effect of educational goal, the effect of student SES on academic achievement (Mathematics: B = 0.02, p < 0.001; Science: B = 0.02, p < 0.001) and the effect of school SES on academic achievement (Mathematics: B = 0.02, p < 0.001; Science: B = 0.01, p < 0.001) were statistically significant. Fig 5 presents the effect sizes from all cycles and countries, categorized by science and mathematics achievement.

The positive effect size of the Student SES * Education Goal interaction indicates that individual socioeconomic conditions provided to students with high educational goals increase their academic achievement more (Mathematics: B = 0.02, p < 0.001; Science: B = 0.02, p < 0.001). In summary, the individual socio-economic conditions provided to students with high educational aspirations improve their academic achievement. The positive effect size of the school SES * Education Goal interaction also demonstrates that students with high educational goals will enhance their academic achievement if they attend a school with high level school SES (Mathematics: B = 0.02, p < 0.001; Science: B = 0.01, p < 0.001).

The effect of the interaction of Mother’s Country of Birth with Student SES on academic achievement (Mathematics: B = 0.01, p > 0.5; Science: B = 0.00, p > 0.5) was statistically insignificant. The effect of the interaction of Mother’s Country of Birth with School SES on academic achievement was significant (Mathematics: B = −0.02, p < 0.001; Science: B = −0.02, p < 0.001). The effect of the interaction of Father’s Country of Birth and Student SES on academic achievement in Mathematics (B = 0.01, p > .05) and the effect of the interaction of Father’s Country of Birth and School SES on academic achievement (B = 0.02, p > .05) were statistically insignificant. In science, the effect of the interaction of Father’s Country of Birth and Student’s SES on academic achievement (B = 0.01, p > .05) was statistically insignificant and the effect of the interaction of Father’s Country of Birth and School SES on academic achievement (B = −0.05, p < .001) was statistically significant. The effect of Student’s Country of Birth and Student’s SES on academic achievement (Mathematics: B = −0.01, p < .05; Science: B = −0.02, p < 0.01) and the effect of Student’s Country of Birth and School SES on academic achievement (Mathematics: B = −0.04, p < 0.001; Science: B = −0.04, p < 0.001) were significant. These findings show that while the mother’s place of birth does not affect the relationship between student SES and academic achievement, the relationship between School SES and academic achievement is higher in favor of students whose mothers are refugees. In other words, the fact that students whose mothers are refugees are educated in schools with high SES levels increases their academic achievement more than those whose mothers are native-born. The father’s country of birth exhibits very little effect on the relationship between SES and academic achievement. The variable “Student’s Country of Birth” indicates the immigrant status of the student. The findings reveal a robust correlation between the immigration status of the student and the impact of student SES on academic achievement, as well as the impact of school SES on academic achievement.

**Fig 6:**
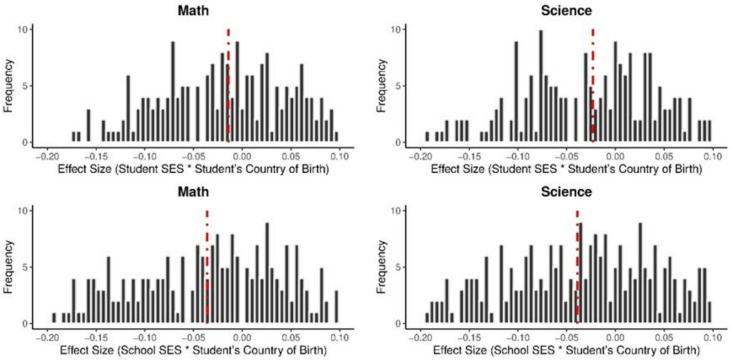
Effect Sizes of Student SES and School SES by Student’s Country of Birth.

The results in Fig 6 suggest that the effect of student SES on academic achievement and the effect of school SES on academic achievement are higher for migrant students. In other words, increasing the individual SES level of migrant students or attending schools with high level school SES increases their academic achievement.

**Fig 7:**
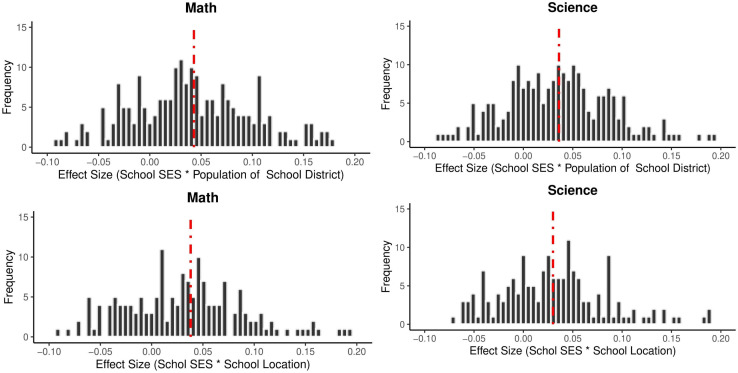
Effect Sizes of Student SES and School SES by Population of School District and School Location.

The variables ‘population of school district’ and ‘location of school’ are school variables and reflect the population of the school district and the location of the school (rural-urban, etc.), respectively. It can be seen that these two variables do not play a moderating role in the relationship between student SES and academic achievement (p > .05). On the other hand, these two variables have a moderating role in the relationship between school SES and academic achievement (p < .001). Fig 7 shows the effect sizes obtained from all cycles and countries separately for science and mathematics achievement.

The graphs show that the effect of school SES on academic achievement is higher in urban and densely populated areas. Therefore, it can be said that the effect of school SES on academic achievement is higher in schools located in urban and densely populated areas, in other words, socio-economically good schools in these regions have higher achievement levels.

#### Subgroup analysis of categorical variables.

Subgroup analysis of categorical variables was conducted using moderator analysis (Table 5). Effect size analysis by year showed that the effect of student SES on academic achievement (Mathematics: Qb (5) = 0.04, p > 0.05; Science: Qb (5) = 0.05, p > 0.05) and the effect of school SES on academic achievement (Mathematics: Qb (5) = 1.08, p > ;0.05; Science: Qb (5) = 1.65, p > 0.05) and school SES on academic achievement (Mathematics: Qb (5) = 1.08, p > 0.05; Science: Qb (5) = 1.65, p > 0.05) did not change across years. The results of the moderator analysis suggest that the increases and decreases in effect sizes by year are not statistically significant.

**Table 5:** Subgroup Analysis of Categorical Variables.

Achievement	Variable	Category	Student Socioeconomic Status (Student SES)								School Socioeconomic Status (School SES)							
			N	Effect Size	se	LB	UB	Q	df	p	N	Effect Size	se	LB	UB	Q	df	p
Mathematics	Year	2003	41	0,180	0,016	0,149	0,211	0,03	5	1,000	41	0,341	0,023	0,296	0,387	1,08	5	0,955
		2007	48	0,186	0,016	0,155	0,216				48	0,325	0,022	0,283	0,368			
		2011	48	0,184	0,017	0,150	0,218				48	0,366	0,024	0,320	0,413			
		2015	44	0,174	0,016	0,144	0,204				44	0,382	0,022	0,338	0,425			
		2019	45	0,178	0,018	0,143	0,212				45	0,410	0,028	0,355	0,465			
		2023	41	0,179	0,023	0,134	0,224				41	0,388	0,027	0,336	0,441			
	Continent	Africa	27	0,065	0,017	0,033	0,098	148,08	5	0,000	27	0,414	0,039	0,337	0,491	31,83	5	0,000
		Asia	121	0,157	0,012	0,134	0,180				121	0,387	0,019	0,349	0,424			
		Europe	81	0,266	0,006	0,253	0,279				81	0,305	0,017	0,272	0,339			
		North America	22	0,196	0,014	0,168	0,224				22	0,384	0,026	0,333	0,436			
		Oceania	11	0,224	0,023	0,179	0,270				11	0,449	0,013	0,423	0,475			
		South America	5	0,118	0,011	0,096	0,140				5	0,514	0,034	0,448	0,580			
	Content Domain	Algebra	263	0,169	0,008	0,153	0,185	38,17	3	0,000	263	0,358	0,016	0,326	0,390	14,38	3	0,002
		Data and Probability	263	0,174	0,010	0,154	0,195				263	0,360	0,017	0,326	0,393			
		Geometry	263	0,169	0,008	0,153	0,186				263	0,347	0,014	0,319	0,374			
		Number	263	0,178	0,008	0,163	0,193				263	0,362	0,013	0,336	0,388			
Science	Year	2003	41	0,189	0,014	0,160	0,217	0,05	5	1,000	41	0,309	0,024	0,262	0,355	1,64	5	0,896
		2007	48	0,198	0,016	0,165	0,230				48	0,304	0,022	0,261	0,347			
		2011	48	0,198	0,018	0,163	0,233				48	0,352	0,023	0,306	0,398			
		2015	44	0,194	0,016	0,162	0,226				44	0,368	0,023	0,323	0,412			
		2019	45	0,197	0,018	0,162	0,233				45	0,401	0,028	0,346	0,455			
		2023	41	0,204	0,023	0,159	0,249				41	0,378	0,025	0,330	0,427			
	Continent	Africa	27	0,064	0,013	0,037	0,090	180,26	5	0,000	27	0,406	0,044	0,320	0,493	30,04	5	0,000
		Asia	121	0,174	0,011	0,152	0,196				121	0,370	0,019	0,332	0,408			
		Europe	81	0,281	0,010	0,261	0,301				81	0,291	0,020	0,253	0,329			
		North America	22	0,238	0,013	0,213	0,264				22	0,363	0,027	0,311	0,415			
		Oceania	11	0,280	0,020	0,241	0,319				11	0,434	0,017	0,402	0,467			
		South America	5	0,142	0,007	0,128	0,156				5	0,484	0,022	0,441	0,527			
	Content Domain	Biology	265	0,195	0,010	0,175	0,216	84,65	3	0,000	265	0,348	0,014	0,319	0,376	6,11	3	0,101
		Chemistry	265	0,180	0,010	0,160	0,200				265	0,343	0,017	0,310	0,375			
		Earth Science	265	0,188	0,008	0,172	0,205				265	0,338	0,016	0,307	0,369			
		Physics	265	0,183	0,008	0,167	0,199				265	0,347	0,016	0,315	0,379			
Cognitive Domain		Knowing	262	0,180	0,008	0,164	0,196	30,06	2	0,000	262	0,371	0,013	0,345	0,396	10,20	2	0,006
		Applying	262	0,178	0,008	0,162	0,193				262	0,368	0,014	0,340	0,396			
		Reasoning	257	0,170	0,010	0,150	0,191				257	0,352	0,018	0,316	0,388			

*Note.* N: Number of Effect Size, se: standard error, LB: lower bound, UB: upper bound.

**Fig 8:**
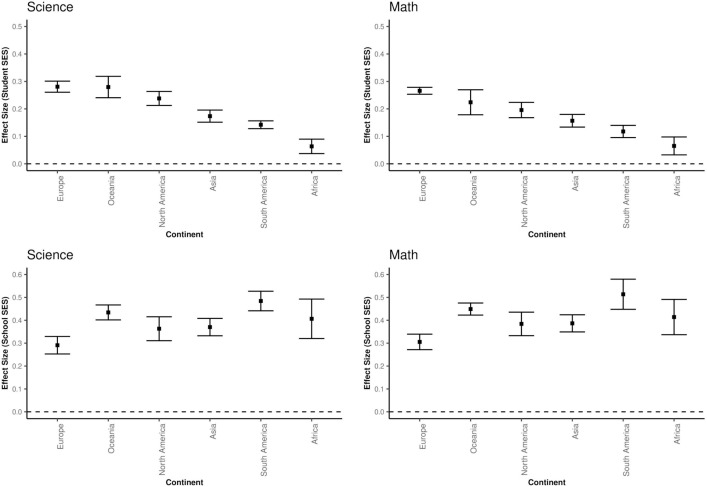
Effect Sizes of Student SES and School SES by Continent.

When analysing the results of the continent variable, it can be seen that in both areas the effect of student SES on academic achievement (Mathematics: Qb (5) = 148.08, p < 0.001; Science: Qb (5) = 180.3, p < 0.001) and the effect of school SES on academic achievement (Mathematics: Qb (5) = 31.8, p < 0.001; Science: Qb (5) = 30.0, p < 0.001) differed by continent. Fig 8 shows the average effect sizes of Student SES and School SES by continent and by academic achievement type.

The ranking of the effect size of Student SES on academic achievement by continent is, from largest to smallest, Europe (Mathematics: b = 0.26; Science: b = 0.28), Oceania (Mathematics: b = 0.22; Science: b = 0. 28), North America (Mathematics: b = 0.20; Science: b = 0.24), Asia (Mathematics: b = 0.16; Science: b = 0.17), South America (Mathematics: b = 0.12; Science: b = 0.14) and Africa (Mathematics: b = 0.07; Science: b = 0.06). The effect size of school SES on academic achievement was South America (Mathematics: b = 0.51; Science: b = 0.48), Oceania (Mathematics: b = 0.45; Science: b = 0. 43), Africa (Mathematics: b = 0.41; Science: b = 0.41), Asia (Mathematics: b = 0.39; Science: b = 0.37), North America (Mathematics: b = 0.38; Science: b = 0.36) and Europe (Mathematics: b = 0.31; Science: b = 0.29). When continents were compared pairwise, the differences between the effect sizes of most continents were statistically significant (see S2 Appendix).

When comparing the mathematics content domain subgroups, there is a significant difference between the content domain groups according to both student SES and school SES (Student SES: Q (3) = 38.1, p < 0.001; School SES: Q (3) = 14.3, p < 0.01). The effect of student SES was largest for the number sub-domain (B = 0.178), followed by data and probability (B = 0.174), geometry (B = 0.169) and algebra (B = 0.169). The difference in effect sizes between these groups, although statistically significant, was quite small. School SES had the largest effect on number (B = 0.362), followed by data and probability (B = 0.360), algebra (B = 0.358) and geometry (B = 0.347). The difference in effect sizes between these groups was statistically significant, but quite small.

When comparing the content domain subgroups of Science, the effect of Student SES on the subgroups was statistically significant (Q (3)=84.65, p < .001), while there was no significant difference between the School SES subgroups (Q = 6.11, p > .05). The effect of student SES was largest for the subfield of biology (B = 0.195), followed by earth science (B = 0.188), physics (B = 0.183) and chemistry (B = 0.180). Although the difference in effect sizes between these groups was statistically significant, it was quite small.

**Fig 9:**
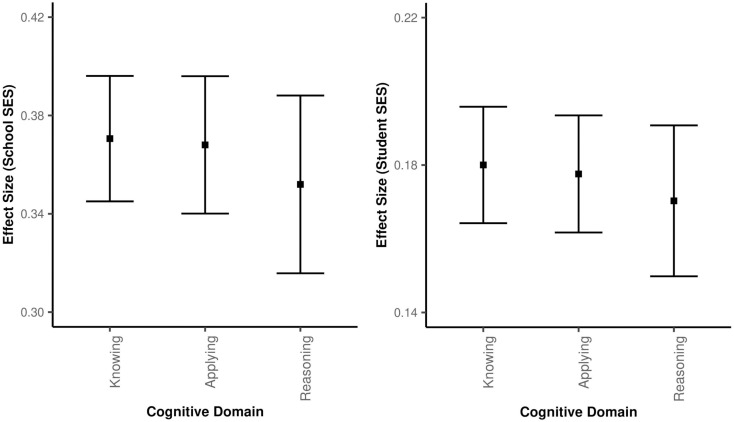
Effect sizes by cognitive domain.

When comparing the cognitive domain subgroups, there were significant differences according to both student SES and school SES (student SES: Q = 30.1, p<0.001; school SES: Q = 10.2, p<0.01). When analysing the effect of student SES on subgroups of cognitive domains, the effect of student SES was largest on the Knowing domain (B = 0.18), followed by Applying (B = 0.18) and Reasoning (B = 0.17). All differences between the sub-domains were statistically significant. Similarly, the effect of school SES on Knowing (B = 0.37) was the largest, followed by Applying (B = 0.36) and Reasoning (B = 0.35). The difference in effect sizes between these groups was statistically significant, but quite small. The effect sizes of the groups are shown in Fig 9.

As the level of the cognitive domain increased, the effect of both student SES and school SES decreased significantly. This finding suggests that the effect of student SES and school SES decreases slightly as the cognitive level of the questions becomes more difficult.

#### Meta regression analysis.

Table 6 shows the results of the meta-regression analysis of the Gini index with student SES and school SES using data from all TIMSS cycles and all countries. The Gini index measures the degree of income inequality in countries. This index takes values between 0 and 1, where 0 indicates a decrease in income inequality and 1 an increase in income inequality.

**Table 6:** Meta regression analysis findings.

Achievement	Variable		N	Coefficient	se	Z	p	LB	UB	Q	df	p
Mathematics	Student SES	intercept	228	0,397	0,038	10,340	0,000	0,322	0,472	33,94	1	0,000
		Gini index	228	−0,592	0,102	−5,826	0,000	−0,791	−0,393			
	School SES	intercept	228	0,154	0,058	2,671	0,008	0,041	0,268	15,65	1	0,000
			Gini index	228	0,602	0,152	3,955	0,000	0,304	0,901		
Science	Student SES	intercept	228	0,426	0,040	10,755	0,000	0,348	0,504	36,4	1	0,000
		Gini index	228	−0,627	0,104	−6,035	0,000	−0,831	−0,424			
	School SES	intercept	228	0,123	0,059	2,097	0,036	0,008	0,238	17,8	1	0,000
			Gini index	228	0,648	0,154	4,220	0,000	0,347	0,949		

*Note.* N: Number of Effect Size, se: standard error, LB: lower bound, UB: upper bound.

**Fig 10:**
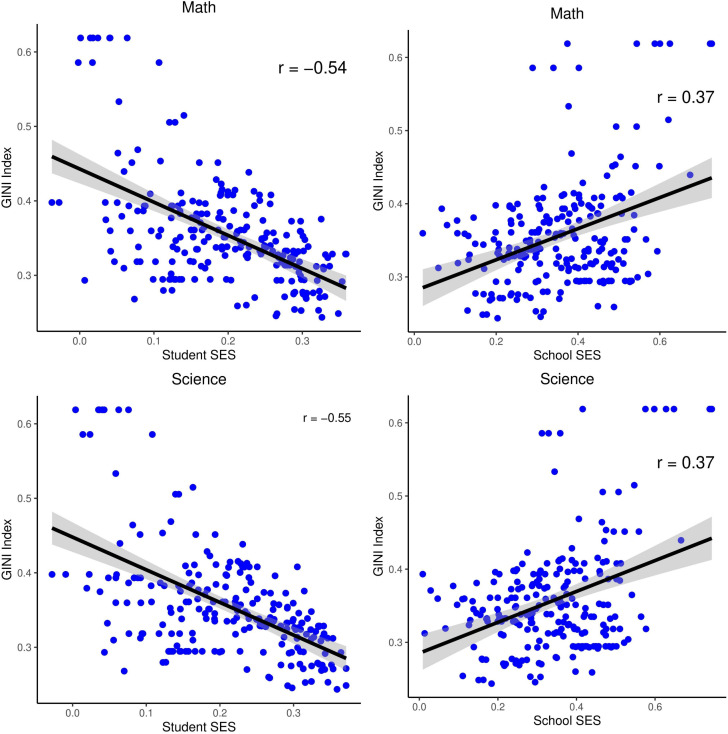
Relationship between Gini Index and Effect Size.

The results show that the effect of the Gini index on student SES is moderate and negative in both mathematics and science (Mathematics: B = −0.59, 95% CI [−0.79, −0.39]; Science: B = −0.63, 95% CI [−0.83, −0.42]). The negative coefficients (β values) indicate that the effect of students’ SES on academic achievement decreases as the Gini index increases. That is, as income inequality in the country increases, the contribution of individual socio-economic status to academic achievement decreases. On the other hand, the effect of the Gini index on student SES is moderate and positive (Mathematics: B = 0.60, 95% CI [0.30, 0.90]; science: B = 0.65, 95% CI [0.35, 0.95]). This finding implies that as income inequality in countries increases, the impact of the socio-economic status of schools on achievement increases. In other words, as income inequality decreases, the effect of individual SES levels on academic achievement becomes more pronounced, while as income inequality increases, schools differentiate in terms of SES level and academic achievement, and students in schools with higher SES levels have higher achievement. Fig 10 shows the relationships between the Gini index – student SES and the Gini index – school SES on a scatterplot (Fig 10).

The results indicate that there is a negative relationship between the Gini index and the effect of student SES on academic achievement (Mathematics: r = −0.54, p < 0.001; Science: r = −0.55, p < 0.001) and a positive relationship between the Gini index and the effect of school SES on academic achievement (Mathematics: r = 0.37, p < 0.001; Science: r = 0.37, p < 0.001).

## Discussion

Socioeconomic status (SES) researchs have become increasingly important in the last century. Researchers have investigated the effects of SES on various aspects of life. In this study, the effects of student SES and school SES on academic achievement are examined in detail using the method of integrative data analysis with data from the TIMSS large-scale assessment for the last 20 years, six cycles and 54 countries. The results show that the impact of SES on academic achievement is strong and stable at the student and school levels.

At the student level, individual SES has a significant impact on academic achievement. Students with higher SES showed better academic achievement in all the cycles analyzed. This finding is consistent with existing literature that emphasises the role of individual socio-economic opportunities on access to the educational process, learning materials and overall academic achievement [7,8,43,73–75]. As students’ economic opportunities increase, factors such as family support, tutoring, access to additional resources and technological infrastructure are thought to support their academic achievement [76,77]. In addition, it can be said that low SES students experience more disadvantages in terms of academic achievement and face structural barriers in the learning process [78,79].

On the other hand, school SES, calculated as the average SES of the student’s peers, was found to have a strong effect on academic achievement. This study found that the academic achievement of students in schools with higher average SES was also higher. The strong effect of school SES on academic achievement is supported by many studies in the literature [11,18,44,80–83]. This suggests that factors such as peer influence, breadth of educational opportunities, access to materials and high quality teaching shape student success. The findings support the impact of the learning environment on academic achievement, as emphasised by ecological models of education [84,85,73]. In schools attended by students from higher socio-economic backgrounds, better equipped teachers, more enriched curricula with more enriching materials, and stronger academic interactions among students are considered to be key factors that increase achievement [86,87].

Another important finding of the study is that school-level SES has a stronger effect than individual SES in many countries. This finding suggests that equal opportunities at the school level are crucial both for ensuring equal opportunities and for raising the academic achievement of the country. The SES of students’ peers is a determinant of students’ academic development. This suggests that education policies should focus not only on individual support programmes, but also on more inclusive and equitable school reforms. There is a limited number of studies in the literature that examine the impact of student and school SES on academic achievement. Some of these studies have found that the impact of school SES on academic achievement is more effective than student SES [48,82,88,89]. Some studies suggest that student SES has a greater impact on academic achievement than school SES [90,91]. In general, it can be said that school SES is one of the effective variables on academic achievement like student SES.

The impact of both student SES and school SES on academic achievement did not vary across cycles (years), but did vary across countries and continents. The impact of student SES on academic achievement was higher in countries in Europe, North America and Oceania, while the impact of school SES on academic achievement was higher in countries in Africa, Asia and South America. There was a small and negative correlation between the effect sizes of school SES and student SES. To explain this difference, the relationship between the Gini index and Student SES and School SES was examined using the meta-regression method. While there was a moderate and negative correlation between student SES and the Gini index, there was a positive and moderate correlation between school SES and the Gini index. This result indicates that there is a relationship between income inequality and student SES and school SES. In other words, it shows that in countries with high income inequality, students with high SES levels are concentrated in successful schools and students with low income levels are educated in unsuccessful schools due to the distorted income distribution caused by income inequality. Similarly, in countries with low income inequality, the SES levels of schools and the SES and achievement distributions between schools are more similar. In these countries, student SES may be more influential than school SES. Therefore, countries, especially those with high level school SES effect sizes, should plan and implement economic and educational policies specific to their own cultural and dynamic contexts to ensure equality of opportunity in education and eliminate income inequality [92–94].

While the effect of student SES on academic achievement was stronger in favour of girls by gender, the effect of school SES on academic achievement was stronger in favour of boys. This finding suggests that increasing the individual SES level of girls will increase their academic achievement more than that of boys. On the other hand, boys attending schools with high SES levels will increase their academic achievement more than girls. This finding shows that the academic achievement of male students is more influenced by the socioeconomic level and social environment of the school. There are studies in the literature that support these findings of the study. de Zeeuw et al. [95] state that girls may benefit more from higher SES, as the difference in educational achievement between boys and girls shrinks in higher SES groups. Eriksson and Lindvall [96] find that the SES*gender interaction favours girls in Western countries, while this interaction favours boys in countries with less gender equality. Thus, there are still debates about the role of gender in the impact of SES on achievement. On the other hand, Cascella [88] argues that in the school SES*gender interaction, the SES composition of the school affects boys’ achievement more than girls’ achievement. Similarly, a number of studies have reported that the school environment for boys drives concept of masculinity in their peer culture and that peer groups encourage or discourage anti-school and academic achievement-oriented behaviors [97,98]. Boys are more likely than girls to respond to a school environment that is focused on learning [99]. Legewie and DiPrete [100] examined the effect of peer SES on academic achievement by gender. The researchers reported that boys were more sensitive to the level of peer SES than girls, and that the learning-oriented school environment increased boys’ academic achievement. The results of the current study support the literature. It was concluded that male students educated in schools with students of high socio-economic level would increase their academic achievement.

The SES achievement effect is stronger for migrant students than for other students. This finding suggests that providing immigrant students with more SES opportunities at the individual or school level increases their academic achievement more than native students. Park and Kyei [101] report that immigrant status has a stronger effect on academic achievement than family SES. Given that immigrants have lower academic achievement [102], the achievement gap can be reduced by providing positive discrimination to immigrants at the individual and school SES levels.

According to the population of the school district and the location of the school, it is concluded that the SES-achievement relationship is stronger in schools with large populations or in urban areas. Boman [103], in his study of Sweden, reports that urban schools may have stronger SES-achievement relationships because the components that make up socio-economic level are higher in large populated or urban areas.

When comparing the sub-groups of the cognitive domain, it was observed that the effects of both student SES and school SES on academic achievement decreased at higher levels of cognitive ability. In other words, the effect of SES on achievement was highest for Knowing, the lowest cognitive level, followed by Applying. The cognitive domain with the lowest SES-achievement effect was Reasoning, the highest level. These results suggest that as the level of the cognitive domain increases, the effect of SES decreases.

Overall, this research shows that socioeconomic status at both the individual and school levels has a significant impact on academic achievement. The findings suggest that while individual SES is important, the socioeconomic status of peers at school may play a larger role in a student’s academic achievement. Education policies should be designed to improve school-level resources and ensure equal educational opportunities, rather than focusing solely on individual support. Given the magnitude of the contribution of high SES schools to student achievement, increasing investment in schools in disadvantaged areas is a critical policy recommendation.

The results of this study can shape future research. In future studies, researchers should further investigate the mechanisms through which school SES affects student achievement. The role of teacher quality, peer relationships, distribution of school resources and funding differences in this effect should be investigated. In particular, long-term longitudinal studies can provide a more comprehensive assessment of changes in socio-economic factors over time and their impact on student achievement. In this study, students’ educational goals were found to be a variable influencing the effect of SES on academic achievement. Studies can be conducted to investigate the relationship between socioeconomic level and students’ higher education goals. In this study, income inequality has a negative relationship with the effect of student SES on academic achievement and a positive relationship with the effect of school SES on academic achievement. Therefore, cross-sectional or longitudinal studies can be conducted to examine the relationship between income inequality and academic achievement. In this study, the SES-academic achievement effect of immigrant students was found to be higher than that of natives. Primary studies can be conducted to examine the SES-academic achievement effect of migrants. On the other hand, the results of this study also provide some recommendations for policy makers. This research shows that SES differences between schools are also quite large. Therefore, policy makers should invest more in disadvantaged students and develop policies to strengthen teacher training programmes and reduce SES differences between schools. The study concludes that there is a negative correlation between individual SES and income inequality and a positive correlation between school SES and income inequality. Particularly in European countries where income inequality is low, SES differences between schools are also low. Therefore, national administrations should develop and implement policies to reduce income inequality in order to reduce SES differences between schools.

## Recommendations

Drawing on the findings of this research, five core policy recommendations can be articulated to guide educational decision-makers. First, since school-level socioeconomic status (SES) exerts a greater influence on student achievement than individual SES, policies should prioritize narrowing inter-school disparities by promoting a fairer allocation of educational resources. Second, schools with predominantly low-SES student populations should be provided with targeted support—such as increased funding, access to well-qualified teachers, improved technological infrastructure, and adequate instructional materials—to help create more supportive learning environments. Third, as the strength and nature of the SES-achievement link differ across national contexts, policy responses should be tailored to specific countries, particularly those with pronounced income inequality, ensuring that interventions are context-sensitive and locally appropriate. Fourth, the results suggest that students from immigrant and refugee backgrounds are especially vulnerable to the negative effects of socioeconomic disadvantage. This highlights the importance of implementing targeted measures in schools serving these communities, including language acquisition programs, psychosocial support services, and initiatives to promote family engagement. Fifth, the enduring nature of SES-related disparities observed throughout two decades of TIMSS assessments indicates the need for long-term strategies. Policymakers should therefore establish systems for the continuous tracking and comprehensive evaluation of interventions aimed at reducing educational inequality.

## Limitations

This study has several limitations that should be acknowledged. First, the analyses were based on secondary data from the TIMSS assessment cycles. While TIMSS provides high-quality, cross-nationally comparable data, the indicators used to measure socioeconomic status (SES) are limited to the variables included in the student and school questionnaires. Thus, the SES index may not fully capture all relevant dimensions of socioeconomic background, potentially leading to measurement bias. Second, the study focused exclusively on eighth-grade students, as the SES index proposed by Broer et al. [41] could not be applied at the fourth-grade level. Therefore, the findings may not be generalizable to younger student populations. Third, although the study covered 54 countries, not all countries participated in every cycle, and the number of cycles varied across countries. This variation may introduce country-specific biases and limit the comparability of results across time and regions. Finally, despite the large and representative sample sizes of TIMSS, the findings may not be fully generalizable to all educational contexts, particularly in non-participating countries or in national systems with unique socioeconomic or cultural structures. Future research should consider using additional datasets, longitudinal designs, and complementary qualitative approaches to further validate and extend these findings.

## Conclusion

This study found that socioeconomic status (SES), at both the individual and school levels, has a significant impact on student achievement across countries and over time. The results show that SES has a similarly significant impact on student achievement over time. The impact of SES on academic achievement varies considerably between countries. In addition, the socioeconomic status of individual students had a significant impact on their academic achievement, and the SES of their peers at school also significantly affected achievement. It is emphasised that education policies should not only focus on individual support programmes, but also on increasing resources at school level and providing more equal opportunities. Given the positive effects on achievement of schools attended by students with high SES levels, increased investment in schools in SES disadvantaged areas is a critical necessity.

The study also found that the effects of student and school SES vary across countries and continents. While the impact of student SES on academic achievement was stronger in regions such as Europe, North America and Oceania, the impact of school SES was more pronounced in regions such as Africa, Asia and South America. This is an important finding that reflects the differences in the educational structure and socio-economic conditions of each region. Moreover, as income inequality increased, differences in SES and achievement between schools became more pronounced, while the importance of individual SES was more pronounced in countries with low income inequality. Accordingly, it is concluded that education policies should be tailored to the unique dynamics of each region.

In conclusion, this research suggests that the SES level of students’ peers is more influential than the individual SES level of the student, and thus the high SES peer environment plays an important role in shaping academic achievement. On the other hand, students with low SES peers had lower achievement. In this context, providing equal opportunities for students and developing policies to address income inequality are of great importance in reducing SES differences between schools in education.

## Supporting information

S1 Appendix Standardized regression coefficients separately for all cycles and all countries. (XLSX)

S2 Appendix Comparing the effect sizes of continents. (DOCX)
