# Supplementary material for: The effect of socioeconomic status on academic achievement: A big data study across countries and time with integrative data analysis
Source: PLoS One. 2025 Oct 31;20(10):e0335485. doi: 10.1371/journal.pone.0335485 (PMC12578351; doi:10.1371/journal.pone.0335485)
Supplement: S2 Appendix — (DOCX) [file pone.0335485.s002.docx]

Appendix – 2. Comparing the Effect Sizes of Continents

| Achievment | Variable | Comparison | t | df | p |
| --- | --- | --- | --- | --- | --- |
| Math | SES | Africa vs Asia | -0,716 | 265 | 0,475 |
| Math | SES | Africa vs Europe | -3,711 | 265 | 0,000 |
| Math | SES | Africa vs North America | -1,571 | 265 | 0,117 |
| Math | SES | Africa vs Oceania | -2,197 | 265 | 0,029 |
| Math | SES | Africa vs South America | 0,266 | 265 | 0,791 |
| Math | SES | Asia vs Europe | -8,443 | 265 | 0,000 |
| Math | SES | Asia vs North America | -1,635 | 265 | 0,103 |
| Math | SES | Asia vs Oceania | -2,633 | 265 | 0,009 |
| Math | SES | Asia vs South America | 1,185 | 265 | 0,237 |
| Math | SES | Europe vs North America | 3,022 | 265 | 0,003 |
| Math | SES | Europe vs Oceania | 1,676 | 265 | 0,095 |
| Math | SES | Europe vs South America | 4,564 | 265 | 0,000 |
| Math | SES | North America vs Oceania | -0,884 | 265 | 0,378 |
| Math | SES | North America vs South America | 2,046 | 265 | 0,042 |
| Math | SES | Oceania vs South America | 2,710 | 265 | 0,007 |
| Math | SES_sch | Africa vs Asia | 6,605 | 265 | 0,000 |
| Math | SES_sch | Africa vs Europe | 7,895 | 265 | 0,000 |
| Math | SES_sch | Africa vs North America | 5,943 | 265 | 0,000 |
| Math | SES_sch | Africa vs Oceania | 4,959 | 265 | 0,000 |
| Math | SES_sch | Africa vs South America | 3,666 | 265 | 0,000 |
| Math | SES_sch | Asia vs Europe | 3,597 | 265 | 0,000 |
| Math | SES_sch | Asia vs North America | 0,058 | 265 | 0,954 |
| Math | SES_sch | Asia vs Oceania | -1,405 | 265 | 0,161 |
| Math | SES_sch | Asia vs South America | -2,152 | 265 | 0,032 |
| Math | SES_sch | Europe vs North America | -1,953 | 265 | 0,052 |
| Math | SES_sch | Europe vs Oceania | -3,298 | 265 | 0,001 |
| Math | SES_sch | Europe vs South America | -3,569 | 265 | 0,000 |
| Math | SES_sch | North America vs Oceania | -1,166 | 265 | 0,244 |
| Math | SES_sch | North America vs South America | -1,909 | 265 | 0,057 |
| Math | SES_sch | Oceania vs South America | -0,926 | 265 | 0,355 |
| Science | SES | Africa vs Asia | -1,306 | 265 | 0,193 |
| Science | SES | Africa vs Europe | -4,361 | 265 | 0,000 |
| Science | SES | Africa vs North America | -2,763 | 265 | 0,006 |
| Science | SES | Africa vs Oceania | -3,712 | 265 | 0,000 |
| Science | SES | Africa vs South America | -0,332 | 265 | 0,740 |
| Science | SES | Asia vs Europe | -8,625 | 265 | 0,000 |
| Science | SES | Asia vs North America | -2,805 | 265 | 0,005 |
| Science | SES | Asia vs Oceania | -4,317 | 265 | 0,000 |
| Science | SES | Asia vs South America | 0,989 | 265 | 0,324 |
| Science | SES | Europe vs North America | 1,916 | 265 | 0,056 |
| Science | SES | Europe vs Oceania | 0,052 | 265 | 0,959 |
| Science | SES | Europe vs South America | 4,429 | 265 | 0,000 |
| Science | SES | North America vs Oceania | -1,349 | 265 | 0,178 |
| Science | SES | North America vs South America | 2,606 | 265 | 0,010 |
| Science | SES | Oceania vs South America | 3,636 | 265 | 0,000 |
| Science | SES_sch | Africa vs Asia | 6,561 | 265 | 0,000 |
| Science | SES_sch | Africa vs Europe | 7,807 | 265 | 0,000 |
| Science | SES_sch | Africa vs North America | 5,957 | 265 | 0,000 |
| Science | SES_sch | Africa vs Oceania | 4,925 | 265 | 0,000 |
| Science | SES_sch | Africa vs South America | 3,813 | 265 | 0,000 |
| Science | SES_sch | Asia vs Europe | 3,469 | 265 | 0,001 |
| Science | SES_sch | Asia vs North America | 0,171 | 265 | 0,865 |
| Science | SES_sch | Asia vs Oceania | -1,439 | 265 | 0,151 |
| Science | SES_sch | Asia vs South America | -1,928 | 265 | 0,055 |
| Science | SES_sch | Europe vs North America | -1,760 | 265 | 0,080 |
| Science | SES_sch | Europe vs Oceania | -3,285 | 265 | 0,001 |
| Science | SES_sch | Europe vs South America | -3,308 | 265 | 0,001 |
| Science | SES_sch | North America vs Oceania | -1,271 | 265 | 0,205 |
| Science | SES_sch | North America vs South America | -1,776 | 265 | 0,077 |
